# Effect of Chemical Surface Texturing on the Superhydrophobic Behavior of Micro–Nano-Roughened AA6082 Surfaces

**DOI:** 10.3390/ma14237161

**Published:** 2021-11-24

**Authors:** Amani Khaskhoussi, Luigi Calabrese, Salvatore Patané, Edoardo Proverbio

**Affiliations:** 1 Department of Engineering, University of Messina, Contrada di Dio Sant’Agata, 98166 Messina, Italy; eproverbio@unime.it; 2 Department of Mathematics and Computer Science, Physical Sciences and Earth Science, University of Messina, Viale F.S. D’Alcontres No. 31, 98166 Messina, Italy; Salvatore.patane@unime.it

**Keywords:** superhydrophobicity, wettability, roughness, surface texturing, fluorine-free silane

## Abstract

Superhydrophobic surfaces on 6082 aluminum alloy substrates are tailored by low-cost chemical surface treatments coupled to a fluorine-free alkyl-silane coating deposition. In particular, three different surface treatments are investigated: boiling water, HF/HCl, and HNO_3_/HCl etching. The results show that the micro-nano structure and the wetting behavior are greatly influenced by the applied surface texturing treatment. After silanization, all the textured surfaces exhibit a superhydrophobic behavior. The highest water contact angle (WCA, ≈180°) is obtained by HF/HCl etching. Interestingly, the water sliding angle (WSA) is affected by the anisotropic surface characteristics. Indeed, for the HF/HCl and the HNO_3_/HCl samples, the WSA in the longitudinal direction is lower than the transversal one, which slightly affects the self-cleaning capacity. The results point out that the superhydrophobic behavior of the aluminum alloys surface can be easily tailored by performing a two-step procedure: (i) roughening treatment and (ii) surface chemical silanization. Considering these promising results, the aim of further studies will be to improve the knowledge and optimize the process parameters in order to tailor a superhydrophobic surface with an effective performance in terms of stability and durability.

## 1. Introduction

Aluminum and its alloys are an important class of engineering materials extensively used in different applications, especially in the transportation, aerospace, and civil industries [1,2,3,4]. Their widespread use is due to their numerous properties, such as good conductivity, low specific weight, and high specific strength. The major drawback of aluminum alloys is the corrosion sensitivity in several environmental conditions, limiting its applicability for structural and functional applications, inducing relevant waste and serious contamination [5,6,7,8]. To avoid this issue and extend their application fields, many researchers proposed the use of a corrosion inhibitor, organic and inorganic coating, chromate-based coating, and anodized layers or polymer duplex coatings [9,10,11,12,13,14,15,16]. However, these methods require complex processes, expensive equipment, and usually toxic chemical agents [17]. Consequently, it is imperative to introduce an alternative strategy, which maximizes the corrosion protection of Al alloys and increases their applicability through an easy, simple, and environmentally friendly surface modification approach. In such a context, a highly promising strategy is to significantly decrease aluminum alloy surface energy in order to reduce its contact with water, promoting its superhydrophobic properties [18,19,20].

The wettability is a relevant solid surface characteristic property [21,22]. Surfaces with a water contact angle (WCA) higher than 150° and a water sliding angle (WSA) less than 10°, defined as superhydrophobic surfaces, have recently attracted intense interest. In nature, several animals and plants show a well-defined superhydrophobic behavior, such as gecko’s feet, water striders, butterfly wings, mosquito eyes, cicada wings, nelumbo nucifera leaves (lotus), marigold petals, rice leaves, rose petals, and nepenthes [23,24,25,26,27]. Their superhydrophobicity derives from the synergistic cooperation between micro- and nano-roughness and low surface energy [28,29,30]. Inspired by the natural superhydrophobic surfaces, scientists have made great efforts to manufacture artificial textured superhydrophobic surfaces. These surfaces can be used in several industrial contexts (e.g., corrosion resistant surface [31,32,33,34] self-cleaning [35,36], anti-icing, anti-biofouling [37], oil–water separation [38,39], and fog condensation [40]). The aforementioned surfaces are usually obtained both by reducing the surface energy and increasing the surface roughness of the metal alloy [41,42,43].

In recent years, different methods have been reported in the literature to develop superhydrophobic surfaces, such as the spray technique [44,45], electrodeposition [46,47], and chemical etching [48,49]. The latter, which consists of a selective and controlled corrosion process, is one of the most promising methods for its industrial application development, due to its simplicity and low manufacturing costs [50,51,52].

Chemical etching provides a feasible approach to produce nano-porous structures with complex morphologies [53]. This approach was effectively applied to address surface texturing of aluminum alloys in order to enhance superhydrophobic behavior. Chen et al. [54] obtained a static water contact angle of 154.8 ± 1.6° on an aluminum substrate’s surface pretreated using chemical etching by using a mixed hydrochloric/acetic acid solution. Analogously, Ruan et al. [55] used myristic acid, lauric acid, and palmitic acid to create a superhydrophobic surface on an Al substrate via the chemical etching method. Rezayi et al. [56] produced a superhydrophobic surface on an aluminum substrate via the chemical etching process followed by nano-ZnO and stearic acid coating. Analogously, Varshney et al. [57] proposed a single- or two-step (chemical etching followed by coating) method to obtain a textured superhydrophobic surface Therefore, several studies have investigated that a superhydrophobic surface with micro-nano rough structure is a key point in order to promote specific performances such as corrosion resistance [58,59], water condensation [60], and self-cleaning [61,62,63,64].

More recently, anisotropic superhydrophobic surfaces with particular liquid–solid adhesion have stimulated huge interest because of their benefits in both fundamental research and industrial applications. By controlling the anisotropic wettability, liquid microdroplets can be driven along the preferred direction according to the designed structure without microvalves, micropumps, or magnetic field actuation, which has a wide-ranging application prospect in the conception and manufacturing of directional fluid control devices and microfluidic systems [65].

So far, a few attempts have been made to assess the complex anisotropic textured wetting surface on metals using only laser-based texturing techniques such as femtosecond and picosecond lasers [66,67]. However, these techniques are inefficient and expensive [49]. Therefore, the identification of a simple and time-saving manufacturing method of a superhydrophobic surface with specific anisotropic behavior for industrial production is still a pending issue. Indeed, further research activities are required in order to better correlate the microstructural texture with the surface properties to optimize the performance and durability of superhydrophobic coated surfaces [68]. A rough micro–nano profile and its possible structural anisotropy may play a key role in the surface properties [66,69]. An interpretation of these phenomena and their correlation to the synthesis process could enhance and extend the applicability of the etching approach to several industrial development contexts.

Considering the above, the aim of the present work is to study the influence of the microstructure on aluminum superhydrophobic surfaces obtained by easy, efficient, industrially feasible, and economical approaches with the purpose of understanding the superhydrophobicity, micro-nano-roughness, and surface anisotropy. The purpose is to improve the knowledge regarding the identification of an easily scalable and feasible approach for the potentially cost-effective manufacturing of superhydrophobic aluminum alloys surfaces.

In particular, in this paper, a two-step strategy was applied: (i) a surface roughening step followed by (ii) a low surface energy coating silanization. Concerning the first step, two different chemical etchings by acid mixture solutions and an immersion in boiling water were used to create rough surfaces. The second step consists of silanization by dip coating, by using an octadecyltrimethoxysilane compound, of the textured surfaces in order to exalt surface hydrophobicity. This silane compound was chosen as an environmentally friendly alternative to the usual fluoroalkylsilane coating. Recently, these fluorine-based compounds were widely investigated thanks to their low surface energy properties highlighting suitable superhydrophobic capabilities, although they induced strict and relevant health and safety issues. Therefore, a fluorine-free coating has been used in this study in order to combine superhydrophobicity and health and environment safety.

The surface texturing on the three resulting etched surfaces was evaluated before and after silanization. The surface morphology, water contact, and sliding angles were also assessed, in both the longitudinal and transversal direction, in order to identify a relationship between the surface microstructure and superhydrophobic behavior of the coated samples.

## 2. Materials and Methods

### 2.1. Materials

Commercially available hot rolled aluminum plates EN AW-6082 T6 (30 mm × 24 mm × 2 mm) were used as substrates. Hydrochloric acid (HCl, 37%) was purchased from Fluka (Buchs, Switzerland). Nitric acid (HNO_3_, 60%) was purchased from Carlo Erba (Cornaredo, MI, Italy). Hydrofluoric acid (HF, 48%) and octadecyltrimethoxysilane (OTMS) (C_21_H_46_O_3_Si, 90%) were procured from Sigma-Aldrich (St. Louis, MI, USA). Toluene was obtained from Riedel-de Haën (Seelze, Germany). Ethanol and acetone were purchased from J.T. Baker (Phillipsburg, NJ, USA). Ultra-pure water from Best Chemical (Vairano Patenora, CE, Italy) was used throughout the experiment.

### 2.2. Synthesis of Superhydrophobic Surfaces

As received aluminum substrates were rinsed ultrasonically with ethanol, acetone, and ultra-pure water and dried at room temperature. A two-step process was applied to create the superhydrophobic surfaces. Initially, the rough texture on the aluminum samples was created by three different procedures (configured on preliminary tests based on literature references [70,71,72,73]):

- HNO_3_/HCl etching: The cleaned sample was dipped in a mixture of HNO_3_ and HCl in an ultra-pure water (1:3:3 ratio in vol.) solution for 60 min.
- HF/HCl etching: The aluminum specimens were dipped for 15 s in a HCl/HF acidic solution (73 vol.% HCl, 5 vol.% HF and 22 vol.% bi-distilled water).
- Boiling water treatment: Aluminum substrates were pretreated in boiling water for 5 min and dried at 70 °C for 60 min.

Before further surface treatments, the etched samples were ultrasonically cleaned and dried at 70 °C for 60 min. Then, the resulting substrates were dipped in 0.1 vol.% octadecyltrimethoxysilane (coded as S18) solution for 10 min in order to reduce their surface energy. Afterward substrates were vertically dried for 3 h at 100 °C for the curing of the silane layer.

Table 1 summarizes the details of the different prepared batches. All samples were coded by using a prefix “Al-”, followed by letters; the first letter indicates the surface roughening process and the second letter indicates if silanization treatment was applied. For example, the Al-WS code represents the aluminum sample immersed in boiling water after silanization.

### 2.3. Sample Characterization

Wettability measurement of the samples was carried out by using Attension Theta equipment (Biolin Scientific, Espoo, Finalnd). A 3 μL liquid droplet was set on the sample surface in a closed box at room temperature. The spherical droplet image was recorded by a micro-camera (CCD - Biolin Scientific, Espoo, Finalnd) and evaluated by shape analyzer PC Attension software included in the instrument. All measurements were replicated 50 times on the whole sample surface for all batches. The sliding angle measurement system (the critical angle where a water droplet starts to slide down from an inclined plate) was configured by using a CCD camera.

The morphology of the different aluminum surfaces before and after coating were checked utilizing scanning electron microscopy (Zeiss Cross-Beam 540, FIB-SEM, Oberkochen, Germany). Furthermore, a VEECO Explorer microscope (Veeco, Plainview, NY, USA) was used to carry out atomic force microscopy (AFM) measurements, in contact mode, through a non-conductive silicon nitride probe model MSCT-EXMT-BF1. The laboratory conditions were controlled at 20 °C and at 60% of relative humidity during all the AFM measurements. The scan rate was set at 0.2 Hz. In addition, the 5 µm × 5 µm scans had a resolution of about 20 nm. All the images were investigated using the AFM software Gwyddion v.2.5 (2018, GNU General Public License, Czech Metrology Institute Brno, Czech Republic).

## 3. Results and Discussion

### 3.1. Surface Morphology

**Figure 1:**
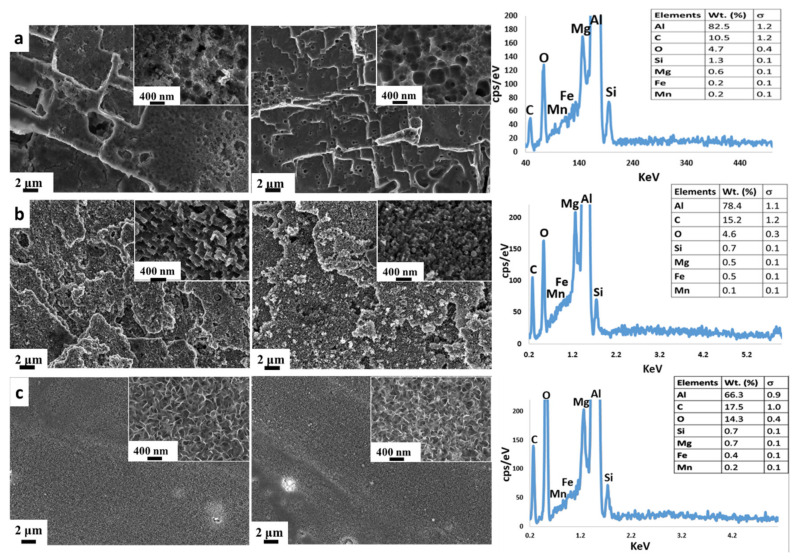
SEM micrographs of the aluminum alloy surfaces before (**left**) and after (**right**) S18 coating deposition. (**a**) Chemical etching in HNO_3_/HCl, (**b**) chemical etching in HF/HCl, and (**c**) boiling water treatment.

At first, a morphological study, carried out by SEM, was used to compare surface textures as obtained by the different surface treatments. Figure 1 shows surface micrographs of the samples soon after etching and after the silanization step. Furthermore, high magnification images of the surfaces are shown in the corner of each micrograph.

No significant differences, as assessed by SEM observation, between the surfaces before and after silanization were observed, thus confirming the very low thickness of the alkylsilane layer deposited on the aluminum surface. Further information can be discussed by comparing the sample morphology observed on the surface sample due to the applied chemical etching:

- HNO_3_/HCl etching: The surface was characterized by a bimodal structure. The coupled action of hydrochloric and nitric acids generated a micro-scale plate-like profile with numerous nano-scale pits (Figure 1a). This morphology is a result of the substrate interaction with this specific acidic solution. It is well known that aluminum alloys react strenuously with hydrochloric acid with a preferential attack of higher energy areas such as dislocations and grain boundaries, usually inducing large, rectangle-shaped pits [74]. However, due to the severe aggressiveness of the solution, the etched areas should be characterized by a non-homogeneous and uniform distribution and size (wider than 1 μm). In such a context, Oh et al. [75] suggested to not use only hydrochloric acid for aluminum etching but a mixture of acidic solution in order to reduce the dissolution rate on the local area and to obtain smaller pits. According to Singh et al. [76], oxidizing acids, such as nitric acid, have less effective corrosive actions on aluminum alloy. The combination of the hydrochloric and nitric acid action can be considered a successful approach to create a hierarchical structure as well as the micro-scale platelet structure that can be seen in the SEM image (Figure 1a).
- HF/HCl etching: The surface morphology of aluminum after the HF/HCl etching and the silane coating steps are shown in Figure 1b. A bimodal morphology can be clearly identified on the surface, i.e., a coral-like structure characterized by the presence of a pixelated substructure at the nanoscale level. In addition, in this case, the microstructure is a consequence of the intrinsic substrate heterogeneity. Indeed, the aluminum alloy has a large number of dislocations and line defects. These local defects are more reactive than other substrate areas in this acidic etchant [75]. Meanwhile, the impurities in the neighborhood of these defects could also magnify the chemical etching reaction. The dissolution phenomena in the aluminum induced by hydrofluoric acid starts after a relatively high immersion time (30–50 min) [77]. According to Straumanis et al. [77], the addition of a small quantity of hydrofluoric acid to other acids allows the electrochemical dissolution of the alloy to be promoted. In addition, HF acid reacts with Si-rich precipitates, favoring a selective dissolution in its neighboring area, thus influencing the large corrosion phenomena induced by a HCl acid solution. The selective dissolution favored by HF, coupled with the wide and general action of HCl, could be considered responsible for the coral-like structure obtained (Figure 1b).
- Boiling water treatment: An environmentally friendly and simple technique to obtain a nanostructured surface was applied. The absence of caustic reagents and special equipment in the whole preparation procedure make it industrially scalable and a cost-effective method. The SEM micrographs of the Al alloy surfaces treated with boiling water are shown in Figure 1c, before and after silanization. At the nano-scale level, the surfaces appeared to be covered by an oxi-hydroxide layer with 3D flower-like structures. This flower-like cluster consists of numerous petals with a thickness of ~30 nm. These contiguous petals overlap and connect to each other, resulting in a complex micro–nano structure. This microstructure is due to the formation of a protective film composed mainly of boehmite, according to the following reaction:

Al + H_2_O → Al_2_O_3_·xH_2_O + H_2_↑ (1)

The generated Al_2_O_3_·xH_2_O reacts with H_2_O to form an AlO(OH) crystal, which is called boehmite [78]. The boehmite formation method is called boehmitage and it is used in industrial engineering to improve the corrosion resistance of Al and its alloys [79]. By using this approach, the further dissolution of the surface is inhibited. This approach was used here to obtain a homogeneous rough surface profile of the aluminum alloy.

### 3.2. Profile Characterization

#### 3.2.1. As-Received

**Figure 2:**
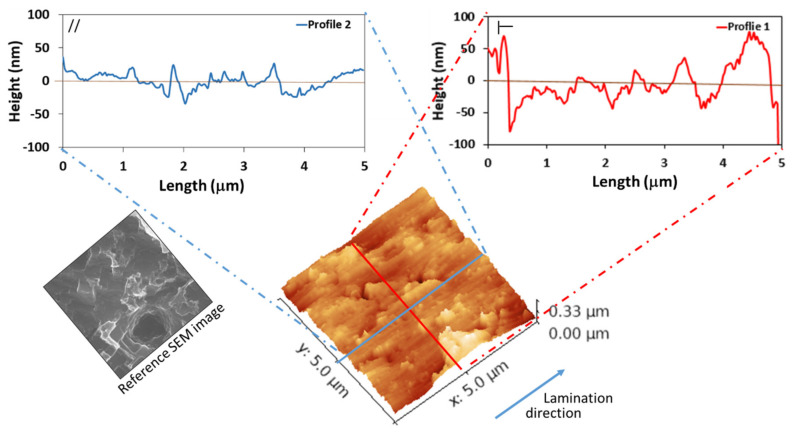
AFM image and typical height distribution histogram of as-received Al surface. **Left**, longitudinal direction; **right**, transversal direction.

Preliminarily, surface profile and roughness of the as-received aluminum alloy surface were investigated by the AFM technique. The analysis was performed both in the longitudinal (parallel to rolling direction) and transversal direction, as shown in Figure 2, in order to assess surface texturing and to discriminate potential morphological anisotropy.

The surface map reveals a relatively smooth surface with numerous parallel grooves (marked grooves at a distance of about 5 µm from each other and light grooves at a distance of about 1 µm) due to the manufacturing technique (lamination). Profile 1 shows the heights in the opposite direction of lamination (transversal direction) where the full gap between the peaks and valleys is clearly higher than that of profile 2 (longitudinal direction).

#### 3.2.2. HNO_3_/HCl Etching

**Figure 3:**
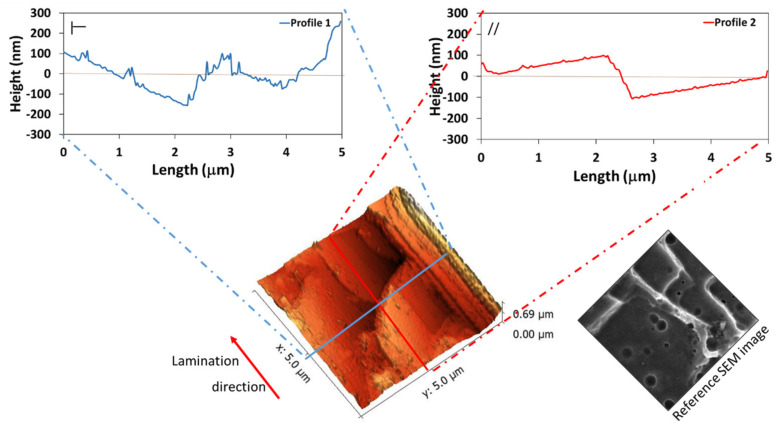
Three-dimensional AFM map and the two direction profiles of Al-NS.

The three-dimensional AFM images and the two direction profiles of aluminum alloy etched with the HNO_3_ and HCl acid mixture solution are shown in Figure 3. It is noticeable that the aluminum alloy substrate became very rough after acid etching. A micro–nano structure was observed on the surface: wide, large, and flat microscale platelets (length of about 2 μm) and nanoscale pits (dimension lower than 150 nm) were identified. This morphological fingerprint was also qualitatively assessed by SEM observations (see Figure 1a and reference SEM image on the right side of Figure 3). The platelets were located at different planes. The steps between two successive platelets were about 200 nm. The nanostructure was constituted by small pits with a diameter of 150 nm nucleated on the flat platelet surfaces. Thus, the roughening treatment with the nitric and hydrochloric acid solution induced the creation of slightly jagged surfaces characterized by numerous steps at the micrometric scale coupled with small pits at the nano-scale.

Moreover, the longitudinal and transverse profiles were not similar. As previously discussed, this is probably due to the initial as-received microstructure anisotropy due to the lamination process (grain flattened and oriented in the rolling direction). The height difference between the peaks and valleys in the lamination direction was about 200 nm. However, this difference was about 300 nm on the direction opposite to the lamination. This morphological anisotropy is attributable to a selective electrochemical attack by the etching solution of crystallographic planes oriented to the lamination direction [80]. However, at this scale, the as-received surface morphology was completely canceled.

#### 3.2.3. HF/HCl Etching

**Figure 4:**
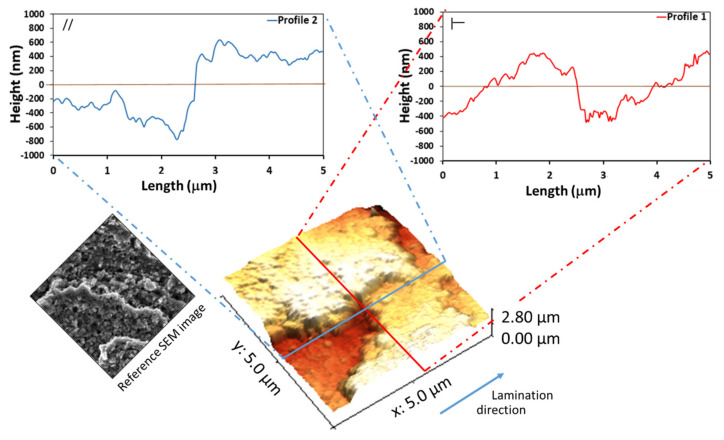
Three-dimensional AFM map and the two direction profiles of Al-FS.

Figure 4 shows the roughness profiles and the AFM map of the Al-FS sample. The visual 3D AFM map shows a surface morphology quite compatible with the SEM images (Figure 1b). In addition, the difference between the longitudinal and transverse profiles persisted somewhat, although topologically the surface profiles in the two directions were similar. In fact, the full gap between the peaks and valleys in the opposite lamination direction was around 1400 nm. Instead, in the lamination direction, this gap was about 1000 nm.

#### 3.2.4. Boiling Water Treatment

**Figure 5:**
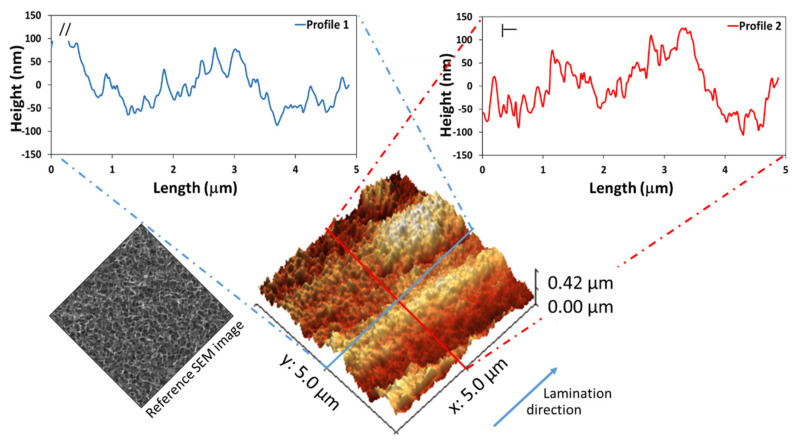
Three-dimensional AFM map and the two direction profiles of Al-WS.

Figure 5 shows the 3D AFM map and the two direction profiles of the Al-WBS. The visual 3D AFM map is morphologically compatible with the SEM images reported in Figure 1c. The roughness profiles are constituted by spheroidal peaks, which are due to the small dimension of flower-like colonies of boehmite that grew during the boiling treatment. The valleys and peaks of the Al-WS profile are not deep. In fact, the full gap between the valleys and peaks was around 200 nm and the root width of each colony was smaller than 15 nm. Lamination marks, even if clearly visible, were less pronounced. The difference between the longitudinal and transverse profiles was less clear, probably due the presence of the boehmite film, which covered the whole surface.

### 3.3. Roughness Statistical Analysis

In order to better understand the evolution of roughness and to highlight the effect of the treatment method and steps, a statistical analysis of the roughness, based on AFM measurements, was carried out and the results summarized in Table 2. Details concerning statistical roughness parameters, listed in the table, are reported in Appendix A.

The results show that the surface roughness on the applied treatment step.

Based on the roughness parameters of the as-received Al alloys, the as-received surface (Al-R) was characterized by a very low roughness (Ra = 10.7 nm; Rms = 16.3 nm). The skewness parameter S ≈ 0 and the kurtosis parameter K ≈ 3 indicate a symmetric and mesokurtic height distribution, typical of Gaussian distribution.

The Ra and Rms of the sample etched with the HNO_3_/HCl acidic mixture solution (Al-N) were around three times higher than the as-received one, pointing out the roughening effect of this type of treatment. The effect of silanization was clearly observed on the surface roughness parameters of Al-NS. In fact, the Ra and Rms decreased by about 50% after the silane deposition. In addition, the skewness, S, reduced from 0.08 to 0.03, indicating a flatter surface. The kurtosis, K, increased from 0.80 to 2.19. This increase was probably due to the reduction of depth and the number of nano-pits, which were filled with silane during coating. Similar Ra and Rms were observed in the Al-W sample. However, the Al-F showed a surface roughness about six times higher that of the other samples (181 nm), indicating a very strong etching effect of the HF/HCl solution on the AA6082 alloy surface.

The Ra and Rms parameters reduced by about 25% after silanization (AL-FS). In addition, the K parameter decreased from 2.01 to 0.18, pointing out a more planar surface than that of the uncoated one. This was also confirmed by the decrease of the S parameter from 1.34 to −0.51. However, the negative S parameters of Al-FS as well as of Al-WS imply that the morphologies of the surfaces were characterized by a large quantity of deep valleys and negative asymmetric distribution. On the other hand, the K parameters of Al-FS and Al-WS were much lower than 3, as they had the values of 0.18 and 0.04, respectively, indicating that these two surfaces had a more jagged and pointed distribution shape than that the normal one.

By a comparison of the three treatments, based on the Ra and the Rms parameters, it can be seen that the Al-F evidenced the highest roughness followed by the Al-N and by the Al-W. In some cases, the surface roughness reduced noticeably after the silanization step. This effect was, however, dependent on the initial morphology of the surface.

This behavior is conferred to the surface morphology, whose roughness has a dual micro- and nano-scale origin. Local cavities and asperities, due to the selective attack of chemical etching, can be identified at the micro-scale level, e.g., on Al-N samples. In this case, the S18 silane molecules may be able to link to the surface in these valleys without hindering issues. This can be justified considering that the silane hydroxyl groups (Si–OH) can easily interact with the hydroxyl groups on the aluminum surface (Al–OH), establishing stable Si–O–Al bonds [81]. Thus, the Al-N sample, which is characterized by quite a flat-wise morphology (platelets with the dimension of at least 2 µm and height of 200 nm), can be readily covered with the homogeneous auto-assembled silane monolayer. The wide-range jagged plateau in the profile had a higher probability to have neighboring hydroxyl groups. Instead, the peaks, because of their narrow and sharp shape, are less susceptible to reaction with the silane molecules due to the limited number of the OH groups in this surface profile area [82].

Subsequently, the silane binds with the surface hydroxyl groups were usually located in the valleys and jagged zones, inducing the reduction of the surface roughness in comparison with the uncoated sample.

In contrast, no roughness change was observed after octadecyltrimethoxysilane coating on the A-W surface. This behavior can be justified by the poor silane mobility. In fact, the silane compound is characterized by a relatively high chemical sensitivity to link on the surface peaks’ extremities, but the reduced mobility limits its capability to enter in the valleys which are very narrow and spiky [83]. Thus, a very thin silane monolayer, with a thickness of about a single molecular size, could be formed in the asperities of the substrate surface, leading to the absence of a clear modification in the roughness after coating.

### 3.4. Wettability

**Figure 6:**
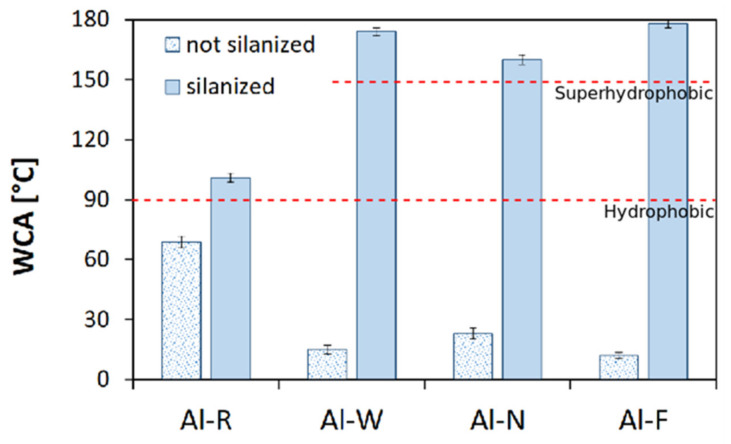
Water contact angles of silanized and not silanized aluminum before and after surface treatments.

In order to better relate the surface behavior to the different treatment methods, the water contact angle (WCA) was measured for all batches (Figure 6); as a reference, the hydrophobicity and superhydrophobicity threshold values were also drawn (dotted red lines).

The wetting behavior of the aluminum alloy surface changed remarkably after each preparation step. In fact, the as-received aluminum alloy, Al-R, showed a hydrophilic behavior typical of a metallic surface with a water contact angle around 69°. After roughening steps, the WCA of all the surfaces decreased, significantly reaching a value lower than 30°. In fact, the water contact angle of Al-N, Al-F, and Al-W were 23° ± 2.72°, 12° ± 1.54°, and 15° ± 2.26° respectively.

This peculiar behavior of the rough surfaces can be explained by the Wenzel state taking into consideration the hydrophilicity of the as-received aluminum alloy surface. The Wenzel state can be defined as a wet contact mode in which the liquid (e.g., water) droplet totally penetrates the surface asperities and the triple-phase air/liquid/solid contact line is stable and regular. For this reason, Wenzel’s approach is also known as homogeneous wetting. Based on the Wenzel model, when the roughness of a hydrophilic surface increases, the water contact angle decreases.

Hence, due to the surface texturing treatment, the aluminum surface, with hydrophilic behavior, apparently becomes markedly hydrophilic. Thus, the rough samples before silanization followed the Wenzel state [84].

After coating with octadecyltrimethoxysilane, the water contact angle of the untreated aluminum increased from 68.7° ± 2.9° to 101° ± 2.2°. This rise of the contact angle was due to the repelling action of the long hydrophobic S18 alkyl molecules [85,86]. When applied on the rough surfaces, the WCA values readily exceeded 150°, thus allowing superhydrophobic surfaces to be obtained. In this case, the water contact angles on the silane coated rough surfaces were inversely proportional to those obtained on the uncoated surfaces. In particular, the sample Al-F, which had the lowest contact angle (12°) after silanization, reached the highest contact angle (179°). This is compatible with the Wenzel model, in which a rough surface enhances the intrinsic surface property, amplifying the hydrophilic or hydrophobic nature of the surface itself [87]. In this context, a significant role was played by the low surface energy and apolar behavior of the alkyl S18 chains. A further effect of the rough micro–nano structures obtained by the chemical etchings was the capability of the surface to trap air bubbles, thus further increasing the hydrophobic behavior of the apolar surface itself and allowing it to move from a Wenzel state to the so-called Cassie–Baxter state in a superhydrophobic behavior [87].

In particular, the highest water contact angle was obtained on the Al-FS sample (average WCA = 179°), while the Al-WS and Al-NS sample surfaces showed an average water contact angle of about 174° and 160°, respectively.

This ranking is in agreement with the AFM measurements, where the roughness observed on the silanized samples was in the order of Al-FS > Al-WS > Al-NS, confirming that the deposition of the octadecyltrimethoxysilane layer on the aluminum alloy surface leads to an improvement of its hydrophobicity due to the presence of hydrophobic alkyl chains. The mechanisms of self-assembled silane monolayer formation on the rough surfaces further promoted the difference of the samples’ wettability behavior. Indeed, the functional long polymer chains of the silane can easily create an organic monolayer characterized by high hydrophobicity. These hydrophobic alkyl chains can have preferential orientations. According to Kulinich et al. [88], alkyl chains molecules orient in an ordered arrangement thanks to the formation of long dipoles and a reciprocal electrostatical interaction by van der Waals forces. However, this effect cannot be obtained if the surface is characterized by limited accessible sites, resulting in a disordered and irregular silane monolayer. This condition leads to a lower hydrophobicity. Thus, it is clear that the surface morphology plays a further role in the surface hydrophobicity by guiding the silanization mechanism.

**Figure 7:**
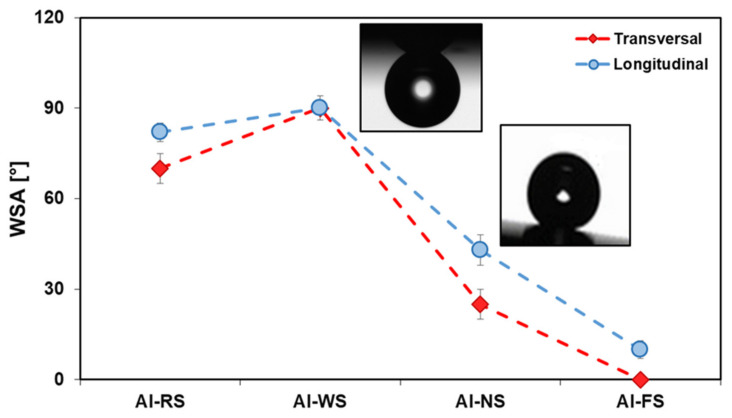
Bidirectional water sliding angles of the as-received aluminum and rough aluminum samples after silanization along the longitudinal and transverse direction to the lamination direction.

Another parameter which allows for the assessment of the wetting behavior of textured surfaces is the water sliding angle. The sliding angle is a measurement of the interaction of the liquid with the surface and is related to the extension of the “wetted” surface area, independent of the hydrophobic behavior, i.e., we can have a hydrophobic surface with high sliding angle (rose petal effect) and a hydrophobic surface with a low sliding angle (lotus effect). Measurements were obtained along both the longitudinal and transversal directions (Figure 7). It is interesting to note that all the samples before silanization (Al-R, Al-N, Al-W, and Al-F) showed a high WSA (90°) (data not reported in Figure 7). While the Al-WS surface showed a WSA > 90° typical of the pure Wenzel state, the Al-FS surface conversely showed a very low WSA almost near to zero, typical of a pure Cassie–Baxter state. An intermediate behavior was observed for the Al-NS surface.

Furthermore, Figure 7 evidences a small but clear influence of the rolling direction on the sliding angles for all batches (with the exception of the Al-WS sample). For these samples, the lowest sliding angles were observed in the opposite direction of the lamination (transverse). Such results are congruent with the roughness analysis. Indeed, the AFM analysis of the bidirectional profiles showed that the full gap between the peaks and valleys in the transverse direction was larger than the longitudinal one, thus promoting the water droplet rolling. A significant evidence of this behavior is reported in the Appendix of this paper. The low water adhesion can be explained by the heterogeneous wetting theory proposed by Cassie. In Cassie’s approach, the liquid does not wet surface valleys because of the trapped air, so the grooves and peaks in the surface profile are in contact with the air and the liquid, respectively [89]. In such a configuration, thanks to the high effective combined action of the silane-induced hydrophobicity and micro–nano roughness, pockets of air can be stably entrapped on the roughness profile in connection with the water/substrate interface, significantly reducing the contact area between the solid and the water droplet and allowing for easier water sliding.

It is interesting to note that, even if the Al-NS and Al-WS surfaces exhibit comparable water contact angles to Al-FS, their adhesion to liquid is different [89,90]. In particular, the water adhesion on the surface of the boiled samples was very high (WSA ~ 90°), indicating that the surface regime was still in a Wenzel state. When the droplet was placed on the superhydrophobic surface, possible van der Waals interactions could still occur between the boehmite layer and the droplet, caused by a non-homogeneous deposition of the silane layer due to the narrow peaks and valleys profile, characterizing the Al-WS sample, and the high density of –OH terminations.

Conversely, for the Al-FS samples, a more homogeneous distribution of the silane layer (as addressed by the SEM images) can be hypothesized by considering the highly superhydrophobic properties. Consequently, the amount of bare metal surface, sensitive to creating van der Waals interactions with water, is limited. At the same time, the intrinsically hierarchical structure of the surface plays a relevant role thanks to the interconnected coral structure that allows for the creation of some air pathways among the trapped air pockets, reducing the negative pressure in them.

Lastly, the intermediate rolling angle (about 25°) characterizing the Al-NS surface can be ascribed to a less interconnected micro–nano porosity on the surface. Indeed, the micro platelets characterized a quite flat surface promoting the solid–liquid interactions. At the same time, the local pits, randomly placed in the surface, allowed for the formation of isolated air pockets. Therefore, the platelets’ morphology favors the Wenzel state and the pits and step-wise structures favor the Cassie–Baxter state. This behavior is defined as an intermediate state between Wenzel and Cassie states [91].

### 3.5. Self Cleaning Behavior

**Figure 8:**
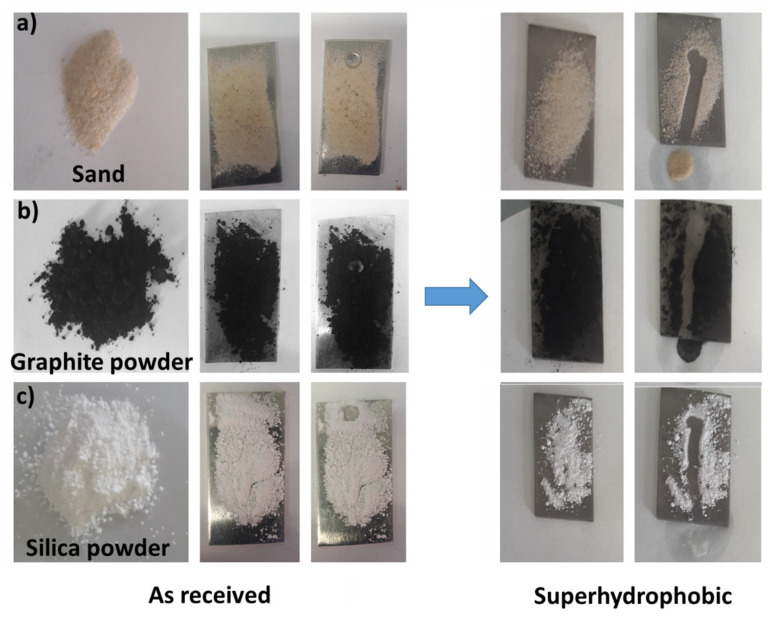
The self-cleaning behavior of the superhydrophobic Al-FS surface using different contaminants: (**a**) sand, (**b**) graphite powder, and (**c**) silica powder. On the left side of the picture, the behavior of the as-received (untreated) aluminum surface is shown as comparison.

A low WSA is fundamental to promote the self-cleaning capability of a surface. A self-cleaning behavior is required for several applications in areas such as textiles, cotton-fabric garments, building construction, lavatories, domestic automobile windows, architectural heritage, and photovoltaic and solar cells [24,27,28,38,49]. Self-cleaning capability can be easily evaluated by a practical water rolling test on randomly spread powder on the superhydrophobic surface. Numerous types of powder as a model dust/contaminant were used (sand, graphite powder, and silica, Figure 8). The powders were distributed on the superhydrophobic surface of the best performing sample (AL-FS), then exposed to water droplets using a disposable syringe. When the water droplets encountered the powder particles, all the types of powder were picked up almost instantaneously and rolled off, leaving a clean surface and confirming the potential suitable self-cleaning capability of this superhydrophobic coating.

In summary, the results indicate that a low surface energy profile coupled with high micro- and nano-rough structures are prone to exhibiting superhydrophobic performances. The values of the statistical parameters effectively describe the surface morphology, i.e., kurtosis and skewness with values around zero and in the range 0.005–2, respectively. Silane deposition significantly modified the wetting behavior. Indeed, all the silanized surfaces evidenced a WCA higher than the threshold for superhydrophobicity (i.e., 150°), but only the Al-FS surface (characterized by a skewness and kurtosis equal to −0.51 and 0.186, respectively) exhibited a sliding angle of almost zero (the water droplet spontaneously rolled off in the transversal direction after deposition) and the highest contact angle, WCA ≈ 180°.

These results indicate that the wettability behavior of an aluminum alloy’s surface can be easily tailored by selecting a specific roughening treatment coupled with proper surface chemical modification. Considering the promising results, further studies will be conducted to improve on the knowledge and optimize the process parameters in order to tailor a superhydrophobic surface with effective performances in terms of stability and durability.

## 4. Conclusions

In this paper, three easy, economical, and timesaving approaches were successfully used to create micro–nano rough surfaces with superhydrophobic behavior. The effects of different texturing parameters on the wettability and the liquid adhesion behavior were assessed in detail. The obtained morphologies were strongly dependent on the used approach. However, all the samples exhibited a hierarchical structure with a high micro- and nano-rough morphology.

Thanks to a silanization step with an environmentally friendly fluorine-free compound (octadecyltrimethoxysilane), in all cases it was possible to reach a WCA higher than 150°. However, only the Al-FS surface (characterized by a skewness and kurtosis equal to −0.51 and 0.186, respectively) exhibited the highest superhydrophobic properties with a WCA ≈ 180° and a WSA ≈ 0°.

Self-cleaning capabilities were successfully confirmed by performing a simplified water rolling test on different types of powder as contaminants (sand, graphite powder, and silica). Accordingly, statistical profile parameters such as skewness and kurtosis, represent suitable indices to support the tailoring processes. The promising results achieved are a stimulus to further develop the proposed synthesis process in terms of synthesis and morphological parameter optimization.

## Figures and Tables

**Table 1:** Sample codes and details.

Samples	Step 1	Step 2
Al-R	–	–
Al-RS	–	S18
Al-N	HNO_3_/HCl etching	–
Al-NS	HNO_3_/HCl etching	S18
Al-F	HF/HCl etching	–
Al-FS	HF/HCl etching	S18
Al-W	Boiling water	–
Al-WS	Boiling water	S18

**Table 2:** Roughness statistic parameters of aluminum alloys at different treatment steps.

Sample	Arith. Roughness Ra (Sa) (nm)	Rms Roughness (Sq) (nm)	Skewness, S	Kurtosis, K
Al-R	10.7	16.3	0.13	3.27
Al-N	33.1	43.1	0.08	0.80
Al-NS	14.9	21.7	0.03	2.19
Al-W	27.5	36.4	0.17	1.41
Al-WS	30.8	37.4	−0.13	0.04
Al-F	181.0	229.	1.34	2.01
Al-FS	138.0	169.8	−0.51	0.18

## Appendix A

*Ra* or *Sa*, arithmetic roughness, is the mean of difference between the height of each single point and the average height of the sample. Thus, this number varies with the interval range. Ra roughness, the most effective roughness commonly adopted in surface engineering, gives a good description of the roughness variation in the surface [92] and may be calculated by the following equation:

(A1) $$Ra=\frac{\sum_{i,j}|a_{i,j}-\langle a\rangle |}{N}$$

where *<a>* is the average height and *N* is the number of points in the studied interval.

The *Rms* roughness is the root mean square roughness. This number varies with the interval range and is sensitive to the height fluctuations. It is given by the following equation:

(A2) $$Rms=\sqrt{\frac{\sum_{i,j}{\left(a_{i,j}-\langle a\rangle \right)}^{2}}{N}}$$

*Ra* and *Rms* parameters are sensitive to the valley and peak variations. The third statistical parameter presented in Table 2 is the surface skewness (*S*). It measures the direction of the asymmetry of the distribution of heights in the sample and it is defined as the average of the first derivative of the surface. This statistical parameter is defined by the expression reported below:

(A3) $$S=\frac{\sum_{i,j}{\left(a_{i,j}-\langle a\rangle \right)}^{3}}{NRms^{3}}$$

The numerical value of surface skewness (*S*) gives us information about the asymmetry direction of the profile distribution in the surface morphology. This statistical parameter is sensitive to the peaks or occasional deep valleys. In fact, a positive surface skewness (*S*) indicates a surface principally characterized by asperities and high peaks and a distribution with “positive asymmetry”. On the other hand, the negative value of surface skewness is related to the large quantity of surface valleys and the distribution has “negative asymmetry”. For a symmetric distribution, the S value is equal to zero and the number of surface valleys is equal to the number of peaks. The S parameter can also be useful to characterize between surfaces having the same Ra and Rms but with different profile shapes.

Finally, the statistic parameter kurtosis (*K*) is a measure of the peakedness (tailedness) of the distribution of heights in the sample by comparing it to the normal distribution. This parameter is given by the following equation:

(A4) $$K=\frac{\sum_{i,j}{\left(a_{i,j}-\langle a\rangle \right)}^{4}}{NRms^{4}}$$

The numerical value of this statistical parameter gives us information about the distribution of heights. In fact, a height distribution is spiked when the kurtosis value is a large positive value (*K* > 3). This spiked distribution is called leptokurtic. *K* = 3 indicates that the height distribution is normal. The statistical term used to describe this distribution is mesokurtic. *K* < 3 is related to distribution of heights skewed above the mean plane (platykurtic distribution) [93].

## References

[1] Wang D., Sun Q., Hokkanen M.J., Zhang C., Lin F.Y., Liu Q., Zhu S.P., Zhou T., Chang Q., He B. (2020). Design of robust superhydrophobic surfaces. Nature.

[2] Aamir M., Giasin K., Tolouei-Rad M., Vafadar A. (2020). A review: Drilling performance and hole quality of aluminium alloys for aerospace applications. J. Mater. Res. Technol..

[3] Varshney D., Kumar K. (2021). Application and use of different aluminium alloys with respect to workability, strength and welding parameter optimization. Ain Shams Eng. J..

[4] Wahid M.A., Siddiquee A.N., Khan Z.A. (2020). Aluminum alloys in marine construction: Characteristics, application, and problems from a fabrication viewpoint. Mar. Syst. Ocean Technol..

[5] Liu K., Zhang M., Zhai J., Wang J., Jiang L. (2008). Bioinspired construction of Mg-Li alloys surfaces with stable superhydrophobicity and improved corrosion resistance. Appl. Phys. Lett..

[6] Chen H., Zhang C., Jia D., Wellmann D., Liu W. (2020). Corrosion Behaviors of Selective Laser Melted Aluminum Alloys: A Review. Metals.

[7] Martínez-Viademonte M.P., Abrahami S.T., Hack T., Burchardt M., Terryn H. (2020). A Review on Anodizing of Aerospace Aluminum Alloys for Corrosion Protection. Coatings.

[8] Calabrese L., Proverbio E., Galtieri G., Borsellino C. (2015). Effect of corrosion degradation on failure mechanisms of aluminium/steel clinched joints. Mater. Des..

[9] Zhang L., Zhou A.G., Sun B.R., Chen K.S., Yu H.Z. (2021). Functional and versatile superhydrophobic coatings via stoichiometric silanization. Nat. Commun..

[10] Idumah C.I., Obele C.M., Emmanuel E.O., Hassan A. (2020). Recently Emerging Nanotechnological Advancements in Polymer Nanocomposite Coatings for Anti-corrosion, Anti-fouling and Self-healing. Surf. Interfaces.

[11] Chidambaram D., Clayton C.R., Halada G.P. (2006). The role of hexafluorozirconate in the formation of chromate conversion coatings on aluminum alloys. Electrochim. Acta.

[12] Johansen H.D., Brett C.M.A., Motheo A.J. (2012). Corrosion protection of aluminium alloy by cerium conversion and conducting polymer duplex coatings. Corros. Sci..

[13] Cai R., Sun M., Chen Z., Munoz R., O’Neill C., Beving D.E., Yan Y. (2008). Ionothermal synthesis of oriented zeolite AEL films and their application as corrosion-resistant coatings. Angew. Chem. Int. Ed..

[14] Chambers B.D., Taylor S.R. (2007). The high throughput assessment of aluminium alloy corrosion using fluorometric methods. Part II—A combinatorial study of corrosion inhibitors and synergistic combinations. Corros. Sci..

[15] Xie Y., Chen H., Shen Y., Tao J., Jin M., Wu Y., Hou W. (2019). Rational Fabrication of Superhydrophobic Nanocone Surface for Dynamic Water Repellency and Anti-icing Potential. J. Bionic Eng..

[16] Melchers R.E. (2019). Predicting long-term corrosion of metal alloys in physical infrastructure. NPJ Mater. Degrad..

[17] Darmanin T., Guittard F. (2013). Superoleophobic surfaces with short fluorinated chains?. Soft Matter.

[18] Zhang D., Wang L., Qian H., Li X. (2016). Superhydrophobic surfaces for corrosion protection: A review of recent progresses and future directions. J. Coat. Technol. Res..

[19] Khaskhoussi A., Calabrese L., Proverbio E. (2019). An Easy Approach for Obtaining Superhydrophobic Surfaces and their Applications. Key Eng. Mater..

[20] Li X., Yu Q., Chen X., Zhang Q. (2021). Microstructures and electrochemical behaviors of casting magnesium alloys with enhanced compression strengths and decomposition rates. J. Magnes. Alloy..

[21] Xiu Y., Xiao F., Hess D.W., Wong C.P. (2009). Superhydrophobic optically transparent silica films formed with a eutectic liquid. Thin Solid Films.

[22] Chen Y.-K., Chang K.-C., Wu K.-Y., Tsai Y.-L., Lu J.-S., Chen H. (2009). Fabrication of superhydrophobic silica-based surfaces with high transmittance by using tetraethoxysilane precursor and different polymeric species. Appl. Surf. Sci..

[23] Bhushan B., Her E.K. (2010). Fabrication of superhydrophobic surfaces with high and low adhesion inspired from rose petal. Langmuir.

[24] Sun T., Feng L., Gao A.X., Jiang L. (2005). Bioinspired Surfaces with Special Wettability. Acc. Chem. Res..

[25] Teisala H., Tuominen M., Kuusipalo J. (2011). Adhesion mechanism of water droplets on hierarchically rough superhydrophobic rose petal surface. J. Nanomater..

[26] Radulović Ž., Porter L.M., Kim T.K., Mulenga A. (2014). Comparative bioinformatics, temporal and spatial expression analyses of Ixodes scapularis organic anion transporting polypeptides. Ticks Tick-Borne Dis..

[27] Watson G.S., Cribb B.W., Watson J.A. (2010). Experimental determination of the efficiency of nanostructuring on non-wetting legs of the water strider. Acta Biomater..

[28] Calabrese L., Khaskhoussi A., Patane S., Proverbio E. (2019). Assessment of Super-Hydrophobic Textured Coatings on AA6082 Aluminum Alloy. Coatings.

[29] Guo Z., Liu W., Su B.L. (2008). Why so strong for the lotus leaf?. Appl. Phys. Lett..

[30] Zhang Y., Chen Y., Shi L., Li J., Guo Z. (2012). Recent progress of double-structural and functional materials with special wettability. J. Mater. Chem..

[31] She Z., Li Q., Wang Z., Tan C., Zhou J., Li L. (2014). Highly anticorrosion, self-cleaning superhydrophobic Ni-Co surface fabricated on AZ91D magnesium alloy. Surf. Coat. Technol..

[32] Isimjan T.T., Wang T., Rohani S. (2012). A novel method to prepare superhydrophobic, UV resistance and anti-corrosion steel surface. Chem. Eng. J..

[33] Liu C., Su F., Liang J., Huang P. (2014). Facile fabrication of superhydrophobic cerium coating with micro-nano flower-like structure and excellent corrosion resistance. Surf. Coat. Technol..

[34] Li X., Liang J., Shi T., Yang D., Chen X., Zhang C., Liu Z., Liu D., Zhang Q. (2020). Tribological behaviors of vacuum hot-pressed ceramic composites with enhanced cyclic oxidation and corrosion resistance. Ceram. Int..

[35] Li W., Kang Z. (2014). Fabrication of corrosion resistant superhydrophobic surface with self-cleaning property on magnesium alloy and its mechanical stability. Surf. Coat. Technol..

[36] Lai Y., Tang Y., Gong J., Gong D., Chi L., Lin C., Chen Z. (2012). Transparent superhydrophobic/superhydrophilic TiO_2_-based coatings for self-cleaning and anti-fogging. J. Mater. Chem..

[37] Xie C., Li C., Xie Y., Cao Z., Li S., Zhao J., Wang M. (2021). ZnO/Acrylic Polyurethane Nanocomposite Superhydrophobic Coating on Aluminum Substrate Obtained via Spraying and Co-Curing for the Control of Marine Biofouling. Surf. Interfaces.

[38] Li J., Shi L., Chen Y., Zhang Y., Guo Z., Su B.L., Liu W. (2012). Stable superhydrophobic coatings from thiol-ligand nanocrystals and their application in oil/water separation. J. Mater. Chem..

[39] Wang C., Yao T., Wu J., Ma C., Fan Z., Wang Z., Cheng Y., Lin Q., Yang B. (2009). Facile approach in fabricating superhydrophobic and superoleophilic surface for water and oil mixture separation. ACS Appl. Mater. Interfaces.

[40] Shang H.M., Wang Y., Limmer S.J., Chou T.P., Takahashi K., Cao G.Z. (2005). Optically transparent superhydrophobic silica-based films. Thin Solid Films.

[41] Ma M., Hill R.M. (2006). Superhydrophobic surfaces. Curr. Opin. Colloid Interface Sci..

[42] Ran M., Zheng W., Wang H. (2019). Fabrication of superhydrophobic surfaces for corrosion protection: A review. Mater. Sci. Technol..

[43] Manoharan K., Bhattacharya S. (2019). Superhydrophobic surfaces review: Functional application, fabrication techniques and limitations. J. Micromanuf..

[44] Wang H., Gao D., Meng Y., Wang H., Wang E., Zhu Y. (2015). Corrosion-resistance, robust and wear-durable highly amphiphobic polymer based composite coating via a simple spraying approach. Prog. Org. Coat..

[45] Wang Z., Chen X., Gong Y., Zhang B., Li H. (2017). Superhydrophobic nanocoatings prepared by a novel vacuum cold spray process. Surf. Coat. Technol..

[46] Zhang B., Zhu Q., Li Y., Hou B. (2018). Facile fluorine-free one step fabrication of superhydrophobic aluminum surface towards self-cleaning and marine anticorrosion. Chem. Eng. J..

[47] Salehikahrizsangi P., Raeissi K., Karimzadeh F., Calabrese L., Proverbio E. (2018). Highly hydrophobic Ni-W electrodeposited film with hierarchical structure. Surf. Coat. Technol..

[48] Kim H.-M., Choi J.-W., Kwon J.-S., Lee C.-H., Kim B. (2019). Super-Hydrophobic Properties of Aluminum Surfaces Synthesized by a Two-Step Chemical Etching Process. J. Nanosci. Nanotechnol..

[49] Lian Z., Xu J., Wang Z., Yu H. (2020). Biomimetic Superlyophobic Metallic Surfaces: Focusing on Their Fabrication and Applications. J. Bionic Eng..

[50] Sun R., Zhao J., Li Z., Qin N., Mo J., Pan Y., Luo D. (2020). Robust superhydrophobic aluminum alloy surfaces with anti-icing ability, thermostability, and mechanical durability. Prog. Org. Coat..

[51] Guo F., Duan S., Wu D., Matsuda K., Wang T., Zou Y. (2021). Facile etching fabrication of superhydrophobic 7055 aluminum alloy surface towards chloride environment anticorrosion. Corros. Sci..

[52] Çakir O. (2008). Chemical etching of aluminium. J. Mater. Process. Technol..

[53] Jiang B., Li M., Liang Y., Bai Y., Song D., Li Y., Luo J. (2016). Etching anisotropy mechanisms lead to morphology-controlled silicon nanoporous structures by metal assisted chemical etching. Nanoscale.

[54] Chen Z., Guo Y., Fang S. (2010). A facial approach to fabricate superhydrophobic aluminum surface. Surf. Interface Anal..

[55] Ruan M., Li W., Wang B., Luo Q., Ma F., Yu Z. (2012). Optimal conditions for the preparation of superhydrophobic surfaces on al substrates using a simple etching approach. Appl. Surf. Sci..

[56] Rezayi T., Entezari M.H., Moosavi F. (2017). The variation of surface free energy of Al during superhydrophobicity processing. Chem. Eng. J..

[57] Varshney P., Mohapatra S.S., Kumar A. (2016). Superhydrophobic coatings for aluminium surfaces synthesized by chemical etching process. Int. J. Smart Nano Mater..

[58] Xiao X., Xie W., Ye Z. (2019). Preparation of corrosion-resisting superhydrophobic surface on aluminium substrate. Surf. Eng..

[59] Salehikahrizsangi P., Raeissi K., Karimzadeh F., Calabrese L., Patane S., Proverbio E. (2018). Erosion-corrosion behavior of highly hydrophobic hierarchical nickel coatings. Colloids Surfaces A Physicochem. Eng. Asp..

[60] Chu F., Wu X. (2016). Fabrication and condensation characteristics of metallic superhydrophobic surface with hierarchical micro-nano structures. Appl. Surf. Sci..

[61] Kumar A., Gogoi B. (2018). Development of durable self-cleaning superhydrophobic coatings for aluminium surfaces via chemical etching method. Tribol. Int..

[62] Attar M.R., Khajavian E., Hosseinpour S., Davoodi A. (2020). Fabrication of micro–nano-roughened surface with superhydrophobic character on an aluminium alloy surface by a facile chemical etching process. Bull. Mater. Sci..

[63] Hooda A., Goyat M.S., Pandey J.K., Kumar A., Gupta R. (2020). A review on fundamentals, constraints and fabrication techniques of superhydrophobic coatings. Prog. Org. Coat..

[64] Sun R., Zhao J., Li Z., Mo J., Pan Y., Luo D. (2019). Preparation of mechanically durable superhydrophobic aluminum surface by sandblasting and chemical modification. Prog. Org. Coat..

[65] Sun D., Böhringer K.F. (2020). An active self-cleaning surface system for photovoltaic modules using anisotropic ratchet conveyors and mechanical vibration. Microsyst. Nanoeng..

[66] Lian Z., Xu J., Yu Z., Yu P., Yu H. (2019). A simple two-step approach for the fabrication of bio-inspired superhydrophobic and anisotropic wetting surfaces having corrosion resistance. J. Alloys Compd..

[67] Samanta A., Wang Q., Shaw S.K., Ding H. (2020). Roles of chemistry modification for laser textured metal alloys to achieve extreme surface wetting behaviors. Mater. Des..

[68] Cao L., Hu H.A., Gao D. (2007). Design and fabrication of micro-textures for inducing a superhydrophobic behavior on hydrophilic materials. Langmuir.

[69] Liang M., Chen X., Xu Y., Zhu L., Jin X., Huang C. (2017). Double-grooved nanofibre surfaces with enhanced anisotropic hydrophobicity. Nanoscale.

[70] Yin L., Wang Y., Ding J., Wang Q., Chen Q. (2012). Water condensation on superhydrophobic aluminum surfaces with different low-surface-energy coatings. Appl. Surf. Sci..

[71] Lee Y., Ju K.Y., Lee J.K. (2010). Stable biomimetic superhydrophobic surfaces fabricated by polymer replication method from hierarchically structured surfaces of al templates. Langmuir.

[72] Feng L., Che Y., Liu Y., Qiang X., Wang Y. (2013). Fabrication of superhydrophobic aluminium alloy surface with excellent corrosion resistance by a facile and environment-friendly method. Appl. Surf. Sci..

[73] Varshney P., Mohapatra S.S., Kumar A. (2017). Fabrication of Mechanically Stable Superhydrophobic Aluminium Surface with Excellent Self-Cleaning and Anti-Fogging Properties. Biomimetics.

[74] Zhang Y., Wu J., Yu X., Wu H. (2011). Low-cost one-step fabrication of superhydrophobic surface on Al alloy. Appl. Surf. Sci..

[75] Oh H.J., Lee J.H., Ahn H.J., Jeong Y., Park N.J., Kim S.S., Chi C.S. (2007). Etching characteristics of high-purity aluminum in hydrochloric acid solutions. Mater. Sci. Eng. A.

[76] Singh D.D.N., Chaudhary R.S., Agarwal C.V. (1982). Corrosion Characteristics of Some Aluminum Alloys in Nitric Acid. J. Electrochem. Soc..

[77] Straumanis M.E., Wang Y.N. (1955). The Rate and Mechanism of Dissolution of Purest Aluminum in Hydrofluoric Acid. J. Electrochem. Soc..

[78] Feng L., Yan Z., Qiang X., Liu Y., Wang Y. (2016). Facile formation of superhydrophobic aluminum alloy surface and corrosion-resistant behavior. Appl. Phys. A Mater. Sci. Process..

[79] Jafari R., Farzaneh M. (2011). Fabrication of superhydrophobic nanostructured surface on aluminum alloy. Appl. Phys. A Mater. Sci. Process..

[80] Zumelzu E., Cabezas C. (1995). Observations on the influence of microstructure on electrolytic tinplate corrosion. Mater. Charact..

[81] Calabrese L., Khaskhoussi A., Proverbio E. (2019). Superhydrophobic behavior of modified AA6082 alloy surfaces. Metall. Ital..

[82] Khaskhoussi A., Calabrese L., Proverbio E. (2020). Superhydrophobic Self-Assembled Silane Monolayers on Hierarchical 6082 Aluminum Alloy for Anti-Corrosion Applications. Appl. Sci..

[83] Rahimi M., Fojan P., Gurevich L., Afshari A. (2014). Effects of aluminium surface morphology and chemical modification on wettability. Appl. Surf. Sci..

[84] Kamusewitz H., Possart W. (2003). Wetting and scanning force microscopy on rough polymer surfaces: Wenzel’s roughness factor and the thermodynamic contact angle. Appl. Phys. A Mater. Sci. Process..

[85] Calabrese L., Bonaccorsi L., Caprì A., Proverbio E. (2016). Effect of silane matrix composition on performances of zeolite composite coatings. Prog. Org. Coat..

[86] Calabrese L., Bonaccorsi L., Capri A., Proverbio E. (2014). Effect of silane matrix on corrosion protection of zeolite based composite coatings. Metall. Ital..

[87] de Gennes P.-G., Brochard-Wyart F., Quéré D., de Gennes P.-G., Brochard-Wyart F., Quéré D. (2004). Dewetting. Capillarity and Wetting Phenomena.

[88] Kulinich S.A., Farzaneh M. (2004). Alkylsilane self-assembled monolayers: Modeling their wetting characteristics. Appl. Surf. Sci..

[89] Lafume A., Quéré D. (2003). Superhydrophobic states. Nat. Mater..

[90] Quére D., Lafuma A., Bico J. (2003). Slippy and sticky microtextured solids. Nanotechnology.

[91] Liu M., Zheng Y., Zhai J., Jiang L. (2010). Bioinspired Super-antiwetting Interfaces with Special Liquid-Solid Adhesion. Acc. Chem. Res..

[92] Horcas I., Fernández R., Gómez-Rodríguez J.M., Colchero J., Gómez-Herrero J., Baro A.M. (2007). WSXM: A software for scanning probe microscopy and a tool for nanotechnology. Rev. Sci. Instrum..

[93] Soliman H.H., Gadelmawla E.S., Koura M.M., Maksoud T.M.A., Elewa I.M. (2002). Roughness parameters. J. Mater. Process. Technol..

